# ^18^F-FDG PET/CT of off-target lymphoid organs in CD19-targeting chimeric antigen receptor T-cell therapy for relapsed or refractory diffuse large B-cell lymphoma

**DOI:** 10.1007/s12149-020-01544-w

**Published:** 2020-11-11

**Authors:** Thorsten Derlin, Christian Schultze-Florey, Rudolf A. Werner, Nora Möhn, Thomas Skripuletz, Sascha David, Gernot Beutel, Matthias Eder, Tobias L. Ross, Frank M. Bengel, Arnold Ganser, Christian Koenecke

**Affiliations:** 1 Department of Nuclear Medicine, Hannover Medical School, Carl-Neuberg-Str. 1, 30625 Hannover, Germany; 2 Department of Hematology, Hemostasis, Oncology and Stem-Cell Transplantation, Hannover, Germany; 3 Department of Neurology, Hannover, Germany; 4 Department of Nephrology, Hannover Medical School, Hannover, Germany

**Keywords:** CAR-T-cell therapy, Lymphoid organs, Outcome, Toxicity, Positron emission tomography

## Abstract

**Objective:**

The interplay between systemic inflammation, activity of lymphoid organs and lymphoma activity in CD19-targeting chimeric antigen receptor (CAR)-T-cell immunotherapy, and its significance for response and toxicity, is not well defined.

**Methods:**

Using serial ^18^F-fluorodeoxyglucose (FDG) positron emission tomography/computed tomography (PET/CT), metabolic parameters of lymphoma and lymphoid organs were analyzed in ten patients receiving Tisagenlecleucel (an autologous CD19 CAR-T cell product) for relapsed or refractory diffuse large B-cell lymphoma. The prevalence and severity of toxicity (e.g., neurotoxicity) were noted.

**Results:**

Achieving remission required early metabolic response (*P* = 0.0476). Early suppression of metabolic activity of lymphoid organs (spleen, *P* = 0.0368; lymph nodes, *P* = 0.0470) was associated with poor outcome. Lymphoma metabolic activity was significantly higher in patients with neurotoxicity (*P* = 0.0489).

**Conclusions:**

Early metabolic changes in lymphoma lesions and off-target lymphoid organs parallel medium-term response to CAR-T-cell therapy. PET can identify patients at risk for severe toxicity.

**Electronic supplementary material:**

The online version of this article (10.1007/s12149-020-01544-w) contains supplementary material, which is available to authorized users.

## Introduction

CD19-targeted chimeric antigen receptor (CAR)-T-cell immunotherapy has shown remarkable efficacy in relapsed and/or refractory (r/r) CD19^+^ B-cell malignancies including diffuse large B-cell lymphoma (DLBCL) [1]. Of note, CAR-T cells expand in vivo and may persist and induce sustained remissions years after initial therapy [2]. However, a substantial fraction of patients will not respond, or relapse, and the mechanisms leading to CAR-T-cell therapy resistance are not fully understood.

Immunological mechanisms may contribute to both resistance and response to CAR-T-cell therapy [2, 3] and also contribute to toxicity. The cytokine release syndrome (CRS) caused by cytokines produced by both activated CAR-T cells and other immune cells such as macrophages mediating a systemic inflammatory response [4] and the immune effector cell-associated neurotoxicity syndrome (ICANS) are common toxicities, treated with immunomodulation [4].

Here, we aimed to further explore the complex interplay between systemic inflammation, activity of bone marrow/off-target lymphoid organs and lymphoma immune cells, and its significance for prediction of response and of toxicity in r/r DLBCL patients undergoing CAR-T-cell therapy. To this end, we applied serial whole-body ^18^F-fluorodeoxyglucose (FDG) positron emission tomography/computed tomography (PET/CT) which is an established clinical molecular imaging technique for imaging of inflammatory and lymphohematopoietic diseases [5], to non-invasively study off-target lymphoid organ activity [6, 7].

## Materials and methods

### Study cohort

From 12 patients with r/r DLBCL which were consecutively referred for PET/CT between March 2019 and February 2020, we retrospectively analyzed ten patients (Table 1). Patients were excluded if showing non-FDG avid indolent lymphoma lesions (*n* = 2). All patients underwent a baseline PET (PET1) before CAR-T-cell therapy, and received PET-based early and late response assessment at + 30 d (PET2, *n* = 7) and + 90 d (PET3, *n* = 5), if still alive and not showing clear evidence of progression on clinical examination or other imaging. Laboratory values including leukocyte count, lymphocyte count and C-reactive protein (CRP) were documented. ^18^F-FDG was administered in compliance with the German Medicinal Products Act, AMG §13.2b, and all subjects provided written informed consent. The institutional review board approved this retrospective analysis (No. 8610-BO-K-2019).

**Table 1:** Patients’ characteristics

Patients *n*	10
Median age (range)—yrs	59 (31–74)
Age ≥ 60 yrs—no. (%)	4 (40)
Gender—male (%) / female (%)	6 (60)/4 (40)
Diagnosis	
Diffuse large B-cell lymphoma	8 (80)
Transformed follicular lymphoma	2 (20)
Disease stage (at diagnosis)	
IA	1 (10)
IIA	2 (20)
IIE	1 (10)
IIIA	3 (30)
IIIB	1 (10)
IVA	2 (20)
International prognostic index (at diagnosis)	1 (0–2)
0	3 (30)
1	3 (30)
2	4 (40)
No. of previous lines of antineoplastic therapy—no. (%)	
2	1 (10)
3	1 (10)
4	3 (30)
≥ 5	5 (50)
Previous autologous hematopoietic stem cell transplantation—no. (%)	5 (50)
Relapsed/Refractory DLBCL—no. (%)	10 (100)
Median time from diagnosis until CAR-T therapy (range)—months	23 (11–197)
Median CAR-T cell dose (range)—cells × 10^8/kg BW	2.8 (1–0-3.50)
CRS—no. (%)	4 (40)
Grade 1–2	3 (30)
Grade ≥ 3	1 (10)
ICAN—no. (%)	4 (40)
Grade 1	1 (10)
Grade 2	3 (30)
Treatment on ICU	3 (30)
Tocilizumab	2 (20)
Steroids	3 (30)
Median follow-up post CAR-T cell therapy (range)—months	4 (0–7)
Progressive disease at last follow-up after CAR-T-cell therapy—no. (%)	7 (70)
Alive at last follow-up after CAR-T-cell therapy—no. (%)	6 (60)
Death due to progressive disease	3 (30)

### CAR-T-cell therapy and assessment of toxicity

In all patients, lymphapheresis lead to successful production of Tisagenlecleucel as autologous CD19 CAR-T-cell product, albeit in two patients’ first attempt failed. All patients underwent lymphodepletion with fludarabine (25 mg/m^2^) and cyclophosphamide (250 mg/m^2^) for three consecutive days prior to infusion of Tisagenlecleucel. Fludarabine and cyclophosphamide were dose adjusted due to reduced kidney function in two patients. Details of CRS and ICANS [4], and CAR-T-cell doses are shown in Table 1. All patients received an extensive neurological work-up prior to CAR-T-cell infusion. During the first 10 days post-CAR-T infusion, patients were daily tested by a neurologist for signs and symptoms of neurotoxicity via clinical neurological examination and cognitive testing using the Montreal-Cognitive-Assessment (MoCA). Patients no. 5, 8 and 9 received PET-guided consolidation radiation therapy after PET2.

### Clinical molecular imaging

PET/CT acquisition and image reconstruction All studies were acquired using a standard PET/CT protocol (Supplemental Data). None of the patients suffered from diabetes. The mean blood glucose level at PET was 92 mg/dL (range 77–112 mg/dL).

**Fig. 1:**
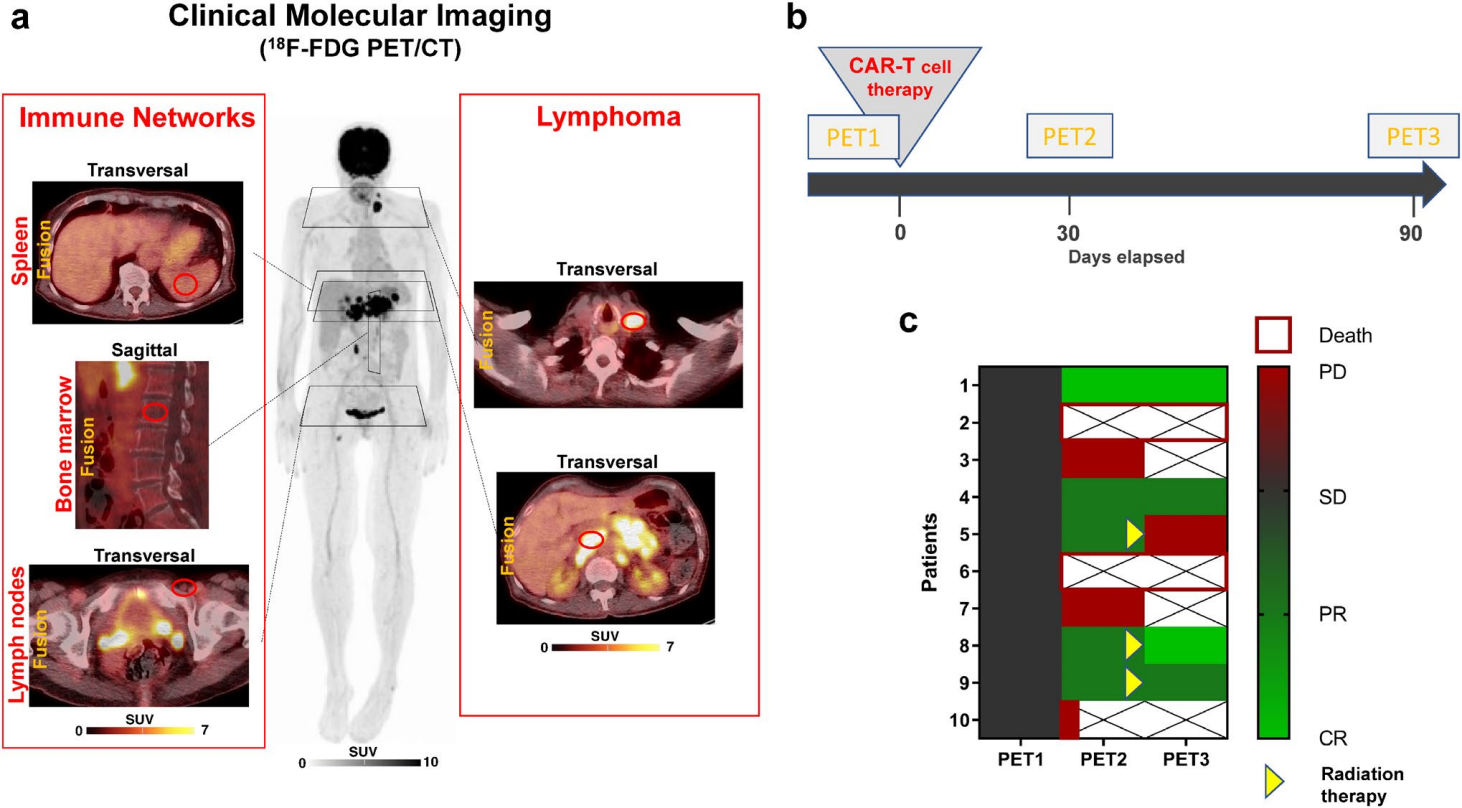
Clinical molecular imaging of the activity of off-target lymphoid organs and lymphoma burden. **a** Metabolic parameters of lymphoid organs and bone marrow (left panel) and lymphoma burden (right panel) were determined using serial ^18^F-FDG PET/CT. **b** Graphical illustration of the time points for serial PET/CT. Patients underwent a baseline scan (PET1) before CAR-T-cell therapy, and early (PET2, + 30d) and late (PET3, + 90d) response assessment scans thereafter, also allowing for assessment of the evolution of metabolic activity. **c** Graphical illustration of response to CAR-T-cell therapy in DLBCL patients, depicted as heat map ranging from complete remission (light green) to progressive disease (red). Patients continued to receive serial PET if still alive and not showing clear evidence of progression on clinical examination or other imaging (crossed out boxes = PET not performed). Remission at Day 90 required early metabolic response at PET2 in all cases (Fisher’s exact test, *P* = 0.0476). Patients no. 5, 8 and 9 received consolidation radiation therapy after PET2

Image analysis and calculation of imaging parameters. PET/CT images were analyzed using a dedicated workstation equipped with a commercial software package (syngo.via; V10B, Siemens Healthcare). All lesions suggestive for lymphoma were noted, and their localization (e.g., lymph node, bone marrow) was recorded. The largest CT diameter of each lesion was recorded, and bulky disease was defined as maximum lesion diameter ≥ 7.5 cm. For assessment of lymphoma metabolic parameters, each lesion was analyzed applying an isocontour volume-of-interest (VOI) including all voxels above 45% of the maximum using a three-dimensional segmentation and computerized volumetric technique. Mean and maximum SUVs (SUV_mean_ and SUV_max_) were measured within all volumes of interest. This measurement also yielded the metabolic tumor volume (MTV). Next, MTV was multiplied by SUV_mean_ of the lesion, yielding the total lesion glycolysis (TLG). Both MTV and TLG of each lesion were summed up to calculate the whole-body MTV and whole-body TLG, respectively. For assessment of bone marrow and lymphoid organ activity (Fig. 1a), the bone marrow signal (averaged SUV_mean_ of 4 VOIs of 2 cm diameter placed within lumbar vertebrae L1 to L4), the spleen signal (averaged SUV_mean_ of 3 VOIs of 2 cm diameter), Waldeyer`s lymphatic ring signal (averaged SUV_max_ of left and right measurements), and the signal from lymph nodes unaffected by lymphoma (averaged SUV_max_ of 4 left and right inguinal and left and right cervical lymph nodes) were determined for each patient at PET1 and PET2, and used for further analyses.

### Assessment of response

Following CAR-T-cell therapy, treatment response was assessed using ^18^F-FDG PET/CT at + 30 d (PET2) and at + 90 d (PET3) according to the Lugano classification [8] criteria. A favourable outcome was defined as achieving partial or complete remission at PET3, whereas an unfavourable outcome was defined as progression, or death.

### Statistical analysis

Categorical variables are presented with absolute and relative frequencies. Continuous variables are expressed as mean ± standard deviation (SD) and range. Normal distribution of data was verified using the Kolmogorov–Smirnov test. For group comparison, an unpaired *t* test (continuous variables) and Fisher’s exact test (categorical variables) were used. Differences in PET metabolic parameters, and their significance for treatment response and toxicity, were analysed. Statistical significance was established for *P* values < 0.05. Analysis was performed using GraphPad Prism^®^ (version 8.3 for Windows; Graphpad Software).

## Results

### Outcomes following CAR-T-cell therapy and significance of early PET response assessment

Two (20%) patients achieved complete remission, two (20%) patients partial remission, four (40%) patients had progressive disease, and two (20%) patients deceased within 3 weeks of therapy. Remission at PET3 required early metabolic response at PET2: all patients achieving a response (PR/CR) at PET3 had already shown an early metabolic response at PET2, whereas only one patient who had an unfavourable outcome had an early response to treatment (*P* = 0.0476) (Fig. 1c).

### Prognostic significance of baseline PET parameters and markers of systemic inflammation

Baseline (PET1) parameters of lymphoma metabolism (*P ≥ *0.1078), lymphoid organ metabolism (*P* ≥ 0.3653), and inflammatory markers (*P ≥ *0.3801) were not significantly different between patients with unfavourable and favourable outcome (Supplemental Table 1). Bulky disease (*P* = 0.0476) was associated with unfavourable outcome.

### Early metabolic changes in lymphoid organs, but not changes in markers of systemic inflammation, are associated with outcome

**Fig. 2:**
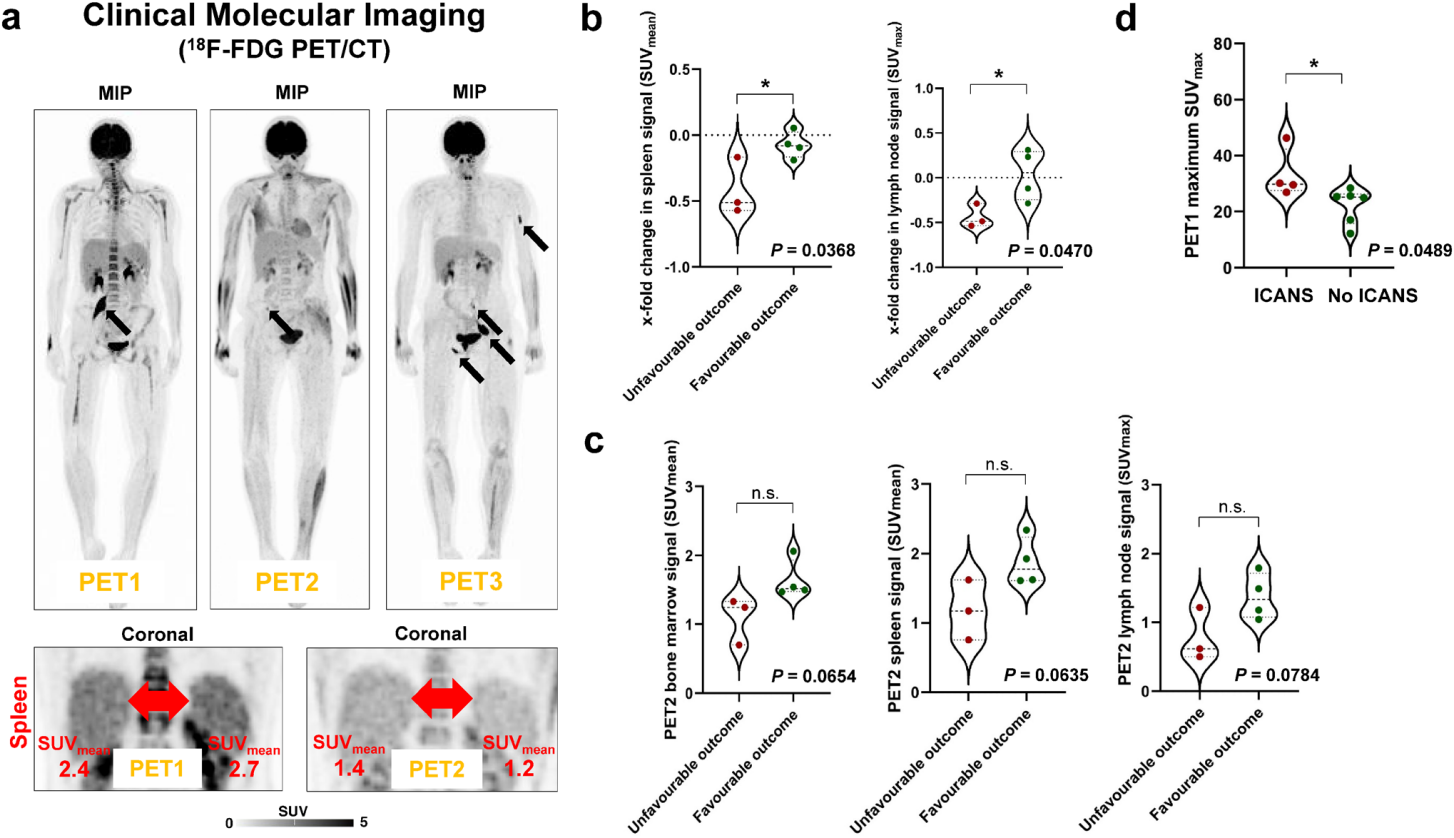
Lymphoid organ metabolism and outcome of CAR-T-cell therapy. **a** Serial ^18^F-FDG PET/CT (upper row) showing metabolically active disease at baseline (PET1), partial remission at PET2, and progressive disease at PET3 (that is, unfavourable outcome). Marked early reduction of spleen activity, both in absolute quantification (SUV_mean_) and relative to liver metabolism (lower row). **b** Marked early reduction in activity of lymphoid organs was associated with worse outcome (spleen signal, *P* = 0.0368; lymph node signal, *P* = 0.0470). **c** Low bone marrow signal and low activity of lymphoid organs early after CAR-T-cell therapy demonstrated a borderline significant association with poor outcome (*P* ≤ 0.0784). **d** Metabolic activity of lymphoma burden was higher in patients developing neurotoxicity (*P* = 0.0489)

Patients with unfavourable outcome demonstrated a significantly higher decrease in both spleen signal (− 42% ± 22% vs. -7% ± 10%; *P* = 0.0368) and lymph node signal (−44% ± 13% vs. + 4% ± 28%; *P* = 0.0470) between PET1 and PET2 (Fig. 2b). By contrast, the change in leukocyte count (*P* = 0.4434) or lymphocyte count (*P* = 0.3054) between baseline and + 30 d was not associated with outcome, and not associated with change in spleen or change in unaffected lymph node signal at PET (*P* ≥ 0.1310 in all cases). Moreover, there was a non-significant trend towards reduced metabolism (SUVs at PET2) in lymphoid organs and bone marrow in patients with unfavourable outcome (spleen, *P* = 0.0635; unaffected lymph nodes, *P* = 0.0784; Waldeyer’s lymphatic ring, *P* = 0.1423; bone marrow, *P* = 0.0654) (Fig. 2c).

### Baseline metabolic activity is associated with development of neurotoxicity

Four (40%) patients developed neurotoxicity. Maximum SUV_max_ at baseline, but not MTV (*P* = 0.1481) or TLG (*P* = 0.1019), was significantly higher in patients who developed neurotoxicity (33.2 ± 8.8 (range 26.9–46.3) vs. 22.3 ± 6.2 (range 12.2–28.4); *P* = 0.0489) (Fig. 2d). No other baseline (PET1) parameter was associated with development of neurotoxicity (*P* ≥ 0.4039 in all cases). 4 (40%) patients developed a CRS. No baseline (PET1) parameter was associated with development of CRS (*P* ≥ 0.0822 in all cases).

In patients having received steroids (*n* = 2) and/or tocilizumab (*n* = 3) for treatment of toxicity, bone marrow signal (*P* = 0.6838), spleen signal (*P* = 0.5688), and lymph node signal (*P* = 0.4802) were not lower at PET2, and the % change between PET1 and PET2 was not different (e.g., change in lymph node signal, *P* = 0.4151).

## Discussion

In this study, we have explored the role of FDG PET-CT in predicting treatment response and toxicity in patients with r/r DLBCL treated with CD19-targeting CAR-T-cell therapy.

Considering the significance of early PET for outcome prediction, we found that achieving remission required early metabolic response at PET2 (*P* = 0.0476) in all cases. In another study including ^18^F-FDG PET scans in seven patients before and 1 month after CAR-T-cell therapy, Shah et al. also demonstrated that the patients with less than complete response all subsequently relapsed [9], underlining the importance of an early complete metabolic response following CAR-T-cell therapy. Indeed, Imber et al. recently reported that in patients with post-CART progression, salvage radiation therapy had to be delivered to sites previously PET-avid pre-CART in the majority of patients [10]. In our cohort, early consolidation radiation therapy after PET2 PR led to CR in one patient, underlining the value of early PET-guided consolidation therapy. In the two other patients receiving image-guided radiotherapy, the outcome was stabilization of PR in one patient, and PD in the other.

We also explored the significance of lymphoid organ activity as determined by molecular imaging, and found that early metabolic changes in lymphoid organs between PET1 and PET2 were associated with unfavourable outcome. Patients with a particularly high lymphoid organ signal decrease (spleen, *P* = 0.0368; lymph nodes, *P* = 0.0470) did not respond to CAR-T-cell therapy. FDG uptake in Waldeyer’s ring was not associated with outcomes, which may be explained by the fact that metabolic activity of Waldeyer’s ring is affected by local immunological response to antigens, and therefore fluctuating. Importantly, in vivo expansion and survival of CAR-T cells after administration are crucial for response, and CAR-T cells have been shown to migrate to the spleen following intravenous injection in preclinical rodent models [11], and to lymph nodes [12]. It remains to be seen if a low spleen or lymph node signal at PET2 indicates limited in vivo expansion of CAR-T cells, or depletion of off-target CD19^+^ B cells, disrupting crucial immune networks for anti-tumor response.

Concerning acute toxicity, the interplay of lymphoma cells, activated CAR-T cells and activated myeloid cells play a crucial role in the pathogenesis of CRS (4). We found that higher lymphoma cell metabolism (SUV_max_) was associated with neurotoxicity (*P* = 0.0489). Importantly, SUV_max_ is directly related to the Ki-67 proliferation index in DLBCL (*r* = 0.7, *P* < 0.01) [13], indicating that patients with high proliferation potential lymphoma may be particularly prone to development of adverse effects. Prospectively, this result could be used for improved risk stratification before CAR-T cell therapy. Of note, Rubin et al. reported cortical and subcortical FDG PET hypometabolism in patients with neurological toxicities [14]. We did not note any noticeable changes in brain metabolism in patients having experienced neurotoxicity, and PET was performed at a later time point (Day 30) in this study. In a study including 19 patients, Wang et al. reported that patients with higher baseline disease burden as determined by FDG PET (both baseline MTV and TLG) had more severe CRS [15]. We did not observe a significant association with disease burden in a smaller patient cohort.

Some limitations should be acknowledged. First, there are inherent limitations associated with the retrospective study design, including a selection bias representing our clinical practice. Second, the small cohort size limited statistical power and precluded a multivariate analysis. Third, we analyzed unaffected bone marrow and lymphoid organs as determined by PET. Although PET is considered the reference standard for detection of lymphomatous involvement of these organs, and DLBCL is usually highly FDG-avid, lymphomatous involvement cannot be excluded with absolute certainty. However, not all lesions can be verified by histology for both practical and ethical considerations, and biopsy itself may deliver false negative results. Finally, a more comprehensive evaluation integrating serial imaging data, immune cell characterization and cytokine profiling would be desirable. We conducted first steps into this direction by including hematology, and this should be seen as a stimulus for subsequent more expansive efforts.

## Conclusion

Serial clinical metabolic imaging allows for elucidating the underlying pathobiology in off-target lymphoid organs in CAR-T-cell therapy, and early metabolic changes in spleen and lymph nodes parallel medium-term response to CAR-T-cell therapy. Moreover, high baseline FDG-avidity of lymphoma was associated with toxicity.

## Electronic supplementary material

Below is the link to the electronic supplementary material.

Supplementary file1 (DOCX 15 KB)
