# Supplementary material for: 18F-FDG PET/CT of off-target lymphoid organs in CD19-targeting chimeric antigen receptor T-cell therapy for relapsed or refractory diffuse large B-cell lymphoma
Source: Ann Nucl Med. 2020 Nov 11;35(1):132–8. doi: 10.1007/s12149-020-01544-w (PMC7796875; doi:10.1007/s12149-020-01544-w)
Supplement: Supplementary file 1 — Supplementary file1 (DOCX 15 KB) [file 12149_2020_1544_MOESM1_ESM.docx]

**Supplemental Data**

**Supplemental Material**

**Supplemental Table 1**

**Supplemental Material**

*PET/CT Acquisition and Image Reconstruction.* All studies were acquired using a Siemens Biograph mCT 128 Flow system (Siemens, Knoxville, USA). Patients received an intravenous injection of 314±12 MBq (range, 285 to 340 MBq) of ^18^F-fluorodeoxyglucose (FDG) following 6h of fasting and confirmation of blood glucose levels < 8mmol/L. Imaging started with a low-dose non-enhanced helical CT (120 kV, mA modulated, pitch 1.2, reconstructed axial slice thickness 5.0 mm) for attenuation correction. Whole-body PET images were subsequently acquired using continuous bed motion at a speed of 1.7 mm/s for head and neck 0.3 mm/s for chest and abdomen and 2.5 mm/s for legs at 1 h post injection (p.i.) after voiding of the bladder. All studies were reconstructed using Ultra HD®, an iterative algorithm combined with time-of-flight and point-spread function information (Siemens Healthcare; 2 iterations, 24 subsets, matrix 200; zoom 1.0; Gaussian filter of 5.0). No contrast material was administered.

**Supplemental Table 1** Metabolic parameters of lymphoma burden and multi-organ immune networks as determined by clinical molecular imaging.

| Time point | Parameter | Outcome | | *P* |
| --- | --- | --- | --- | --- |
|  |  | Unfavourable | Favourable |  |
| PET1 | Lymphoma burden |  |  |  |
|  | MTV (cm^3^) | 172±148 (11 to 401) | 34±24 (12 to 58) | 0.1078 |
|  | TLG (cm^3^) | 2457±2535 (134 to 6427) | 402±357 (43 to 770) | 0.1529 |
|  | Maximum SUV_max_ | 28.7±9.7 (17.0 to 46.3) | 23.5±7.9 (12.2 to 30.1) | 0.4008 |
|  | Immune networks signal |  |  |  |
|  | Bone marrow (SUV_mean_) | 1.7±1.3 (0.8 to 4.2) | 1.7±0.7 (0.7 to 2.4) | 0.9968 |
|  | Spleen (SUV_mean_) | 1.9±0.6 (1.2 to 2.7) | 2.1±0.6 (1.7 to 2.9) | 0.6053 |
|  | WLR (SUV_max_) | 2.8±0.9 (2.0 to 4.0) | 3.5±1.6 (2.0 to 5.6) | 0.3653 |
|  | LN (SUV_max_) | 1.3±0.3 (0.8 to 1.7) | 1.4±0.2 (1.1 to 1.7) | 0.5975 |
|  |  |  |  |  |
| PET2 | Lymphoma burden |  |  |  |
|  | MTV (cm^3^) | 275±397 (2 to 731) | 12±17 (1 to 38) | 0.2292 |
|  | TLG (cm^3^) | 2761±3852 (4 to 7162) | 28±37 (3 to 82) | 0.2018 |
|  | Maximum SUV_max_ | 17.7±12.1 (4.3 to 27.6) | 6.4±2.6 (2.9 to 8.6) | 0.1198 |
|  | Immune networks signal |  |  |  |
|  | Bone marrow (SUV_mean_) | 1.1±0.3 (0.7 to 1.3) | 1.6±0.3 (1.5 to 2.1) | 0.0654 |
|  | Spleen (SUV_mean_) | 1.2±0.4 (0.8 to 1.6) | 1.9±0.3 (1.6 to 2.3) | 0.0635 |
|  | WLR (SUV_max_) | 1.9±0.5 (1.4 to 2.4) | 3.6±1.6 (2.3 to 6.0) | 0.1423 |
|  | LN (SUV_max_) | 0.8±0.4 (0.5 to 1.2) | 1.4±0.3 (1.0 to 1.8) | 0.0784 |
|  |  |  |  |  |

Values are mean±standard deviation (range). LN – lymph node(s); MTV – metabolic tumor volume; SUV – standardized uptake value; TLG – total lesion glycolysis; WLR – Waldeyer`s lymphatic ring
